# Carbon monoxide poisoning: a prediction model using meteorological factors and air pollutant

**DOI:** 10.1186/s12919-021-00206-7

**Published:** 2021-03-02

**Authors:** Hai-Lin Ruan, Wang-Shen Deng, Yao Wang, Jian-Bing Chen, Wei-Liang Hong, Shan-Shan Ye, Zhuo-Jun Hu

**Affiliations:** 1 Department of Emergency, Liuzhou Worker’s Hospital, The Fourth Affiliated Hospital of Guangxi Medical University, Liuzhou, Guangxi 545005 PR China; 2 Guangxi Liuzhou Meteorological Bureau, Liuzhou, Guangxi 545001 PR China; 3 Guangxi Liuzhou Environmental Protection Bureau, Liuzhou, Guangxi 545006 PR China; 4 Department of Respiration, Liuzhou Worker’s Hospital, The Fourth Affiliated Hospital of Guangxi Medical University, Liuzhou, Guangxi 545005 PR China

**Keywords:** CO poisoning, Meteorological factors, Air pollutant levels, Prediction

## Abstract

**Background:**

While the influence of meteorology on carbon monoxide (CO) poisoning has been reported, few data are available on the association between air pollutants and the prediction of CO poisoning. Our objective is to explore meteorological and pollutant patterns associated with CO poisoning and to establish a predictive model.

**Results:**

CO poisoning was found to be significantly associated with meteorological and pollutant patterns: low temperatures, low wind speeds, low air concentrations of sulfur dioxide (SO_2_) and ozone (O_3_8h), and high daily temperature changes and ambient CO (r absolute value range: 0.079 to 0.232, all *P* values < 0.01). Based on the above factors, a predictive model was established: “logitPj = aj - 0.193 * temperature - 0.228 * wind speed + 0.221 * 24 h temperature change + 1.25 * CO - 0.0176 * SO_2_ + 0.0008 *O_3_8h; j = 1, 2, 3, 4; a1 = -4.12, a2 = -2.93, a3 = -1.98, a4 = -0.92.” The proposed prediction model based on combined factors showed better predictive capacity than a model using only meteorological factors as a predictor.

**Conclusion:**

Low temperatures, wind speed, and SO_2_ and high daily temperature changes, O_3_8h, and CO are related to CO poisoning. Using both meteorological and pollutant factors as predictors could help facilitate the prevention of CO poisoning.

**Supplementary Information:**

The online version contains supplementary material available at 10.1186/s12919-021-00206-7.

## Background

Carbon monoxide (CO) poisoning is the main cause of emergency department admissions, and it is a leading cause of death from poisoning worldwide [1]. Studies showed that CO poisoning affects 50,000 people per year in the United States [2] and that most cases are unintentional [3]. Domicile-related CO poisoning mostly occurs in night and is difficult to detect [4]. Hence, the development of effective prediction for CO poisoning is of great public health importance. In China, domicile-related CO poisoning prevails with the main sources of CO being biomass and coal stoves [5].

Many studies have explored how meteorological patterns influence the prevalence of CO poisoning, which largely occurs in winter and is associated with low meteorological temperatures, low barometric pressure and low wind speeds [6]. Weather patterns influence pollutant distributions, and their combination might thus play important roles in the initiation of domicile-related CO poisoning [7]. As local real-time meteorological and pollutant data are easy to obtain, they may serve as effective predictors. However, few data are available on pollutant patterns related to CO poisoning. Over the past two decades, several studies have been published on many aspects of CO poisoning in the realms of pathophysiology, diagnosis, and clinical management along with evidence-based recommendations for optimal clinical practice [4]. Though many improvements have been made, CO poisoning remains a serious problem worldwide and especially in developing countries [8]. Developing effective forecasting methods could help better prevent the poorly understood and collectively appearing results of poisoning, but their prediction has been largely not explored and their effects remain unclear.

Publications on CO poisoning and associated deaths occurring in mainland China are very limited [8, 9], and there is a paucity of work on how meteorological and pollutant patterns influence CO poisoning. Hence, the aim of the present study was to characterize meteorological and pollutant patterns associated with CO poisoning in Liuzhou (a city in southern China) and to explore a prediction model for this issue.

## Results

### Monthly distribution of CO poisoning

**Fig. 1:**
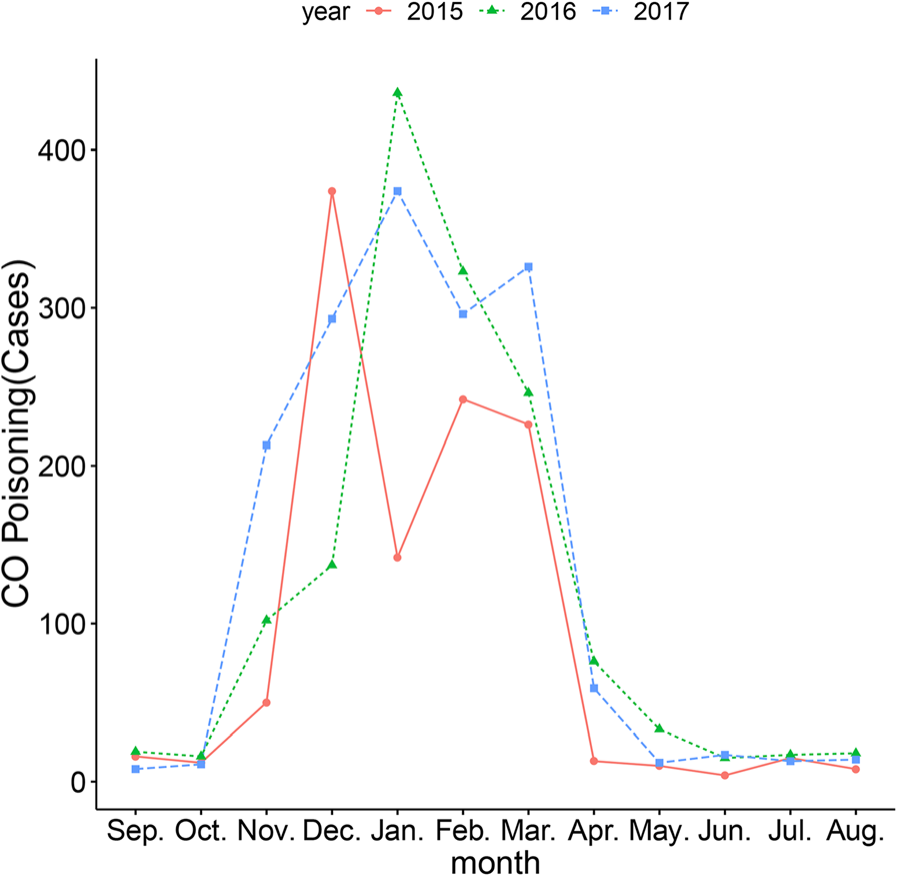
Monthly distribution of CO poisoning from 2015 to 2017 in Liuzhou

The cases of CO poisoning showed a pattern of annual cyclicity with a peak occurring from December to March (Fig. 1) and with cases ranging from 0 to 500 per month. The cases of CO poisoning in January of 2016 and 2017 was at top, but an abruptly decrease occurred in that month of 2015 year. Cases of CO poisoning recorded from December to March account for more than 80% of all cases occurring from 2015 to 2017.

### Missing value processing and air pollutant levels

The days with missing value of pollutant factor (approximately 0.2–0.5%) were excluded from the analysis. From 2015 to 2017, the daily mean concentrations of SO_2_, NO_2_, CO, and O_3_8h were 24.82 μg/m3, 26.11 μg/m3, 1.048 mg/m3, and 87.51 μg/m3, respectively, and daily mean concentrations of PM_10_ and PM_2.5_ were 74.98 and 53.13 μg/m3, respectively (Additional file 1: Table S1). Overall, air pollutant concentrations were low, though some were in a moderate level according to China Ambient Air Quality Standards (GB 3095–2012).

### Correlations between meteorological and pollutant parameters and the occurrence of CO poisoning

Relationships between the occurrence of CO poisoning and meteorological parameters (temperature, air pressure, humidity, wind direction and wind speed) and pollutant parameters (SO_2_, NO_2_, PM_10_, PM_2.5_, CO, and O_3_8h) were analyzed. We found that CO poisoning was significantly and inversely associated with temperatures, 24-h atmospheric pressure changes, wind speeds and ozone (O_3_8h) (r ranges: − 0.162 to − 0.409, all *Ps <* 0.001) and positively associated with 24-h temperature changes, atmospheric pressure, humidity, and pollutants (SO_2_, NO_2_, PM_2.5,_ PM_10_, and CO) (r ranges: 0.085 to 0.371, all *Ps <* 0.01) (Table 1 and Additional file 1: Table S2). Overall, the association of temperature-related indices with CO poisoning was stronger among meteorology indices, while it was stronger for NO_2_ (followed by CO and PM_2.5_) among pollutant indices.

**Table 1:** Pearson correlation between CO poisoning admission and meteorological parameters

	Mean temperature	24-h temperature difference	Mean atmospheric pressure	24-h atmospheric pressure change	Humidity	Maximum wind direction	Mean wind speed	CO poisoning cases
Mean temperature	1							
24-h temperature change	0.164†	1						
Mean atmospheric pressure	−0.881†	− 0.123†	1					
24-h atmospheric pressure change	− 0.095‡	− 0.546†	0.200†	1				
Humidity	−0.023	− 0.04	− 0.228†	− 0.139†	1			
Maximum wind direction	−0.069‡	−0.137†	0.030	0.123†	0.117†	1		
Mean wind speed	0.183†	−0.166†	− 0.155†	0.245†	− 0.257†	0.143†	1	
CO poisoning cases	−0.409†	0.240†	0.353†	−0.162†	0.085‡	0.049	−0.269†	1

*Abbreviation*: *CO* Carbon monoxide

†*P*-value for correlation < 0.001

‡*P*-value for correlation between 0.001 and 0.05

A partial correlation analysis showed that temperature, wind speed, SO_2_ and O_3_8h (R ranges: − 0.079 to − 0.152, all *Ps <* 0.01) were inversely correlated with CO poisoning incidence, and 24-h temperature change and air CO concentration (R ranges: 0.098–0.232, all *Ps <* 0.01) were positively correlated with CO poisoning incidence (Table 2). No significant correlations were found for other indices. Thus, in further prediction analysis, these six indices with significant partial correlations with CO poisoning were used for predictors.

**Table 2:** Partial correlation between CO poisoning admission and meteorological and environmental factors

Factors	Coefficient	*P*-value
Mean temperature, °C	**−0.152**	**< 0.001**
24-h temperature change, °C^a^	**0.232**	**< 0.001**
Mean atmospheric pressure, hPa	−0.004	0.882
24-h atmospheric pressure change, hPa^a^	0.030	0.326
Humidity, %	−0.022	0.464
Maximum wind direction, °	0.057	0.059
Mean wind speed, m/s	**−0.079**	**0.009**
SO_2_, ug/m^3^	**−0.112**	**< 0.001**
NO_2_, ug/m^3^	0.021	0.488
PM_10_, ug/m^3^	0.046	0.134
CO, mg/m3	**0.098**	**0.001**
O_3_8h, ug/m^3^	**−0.106**	**< 0.001**
PM_2.5_, ug/m^3^	−0.010	0.734

Data are shown as partial correlation coefficient (significance)

*Abbreviations*: *CO* Carbon monoxide, *NO*_*2*_ Nitrogen dioxide, *O*_*3*_*8h* Ozone, *PM* Particulate matter, *SO*_*2*_ Sulfur dioxide

^a^The value was calculated as difference on the second day minus the value on the first day

**Fig. 2:**
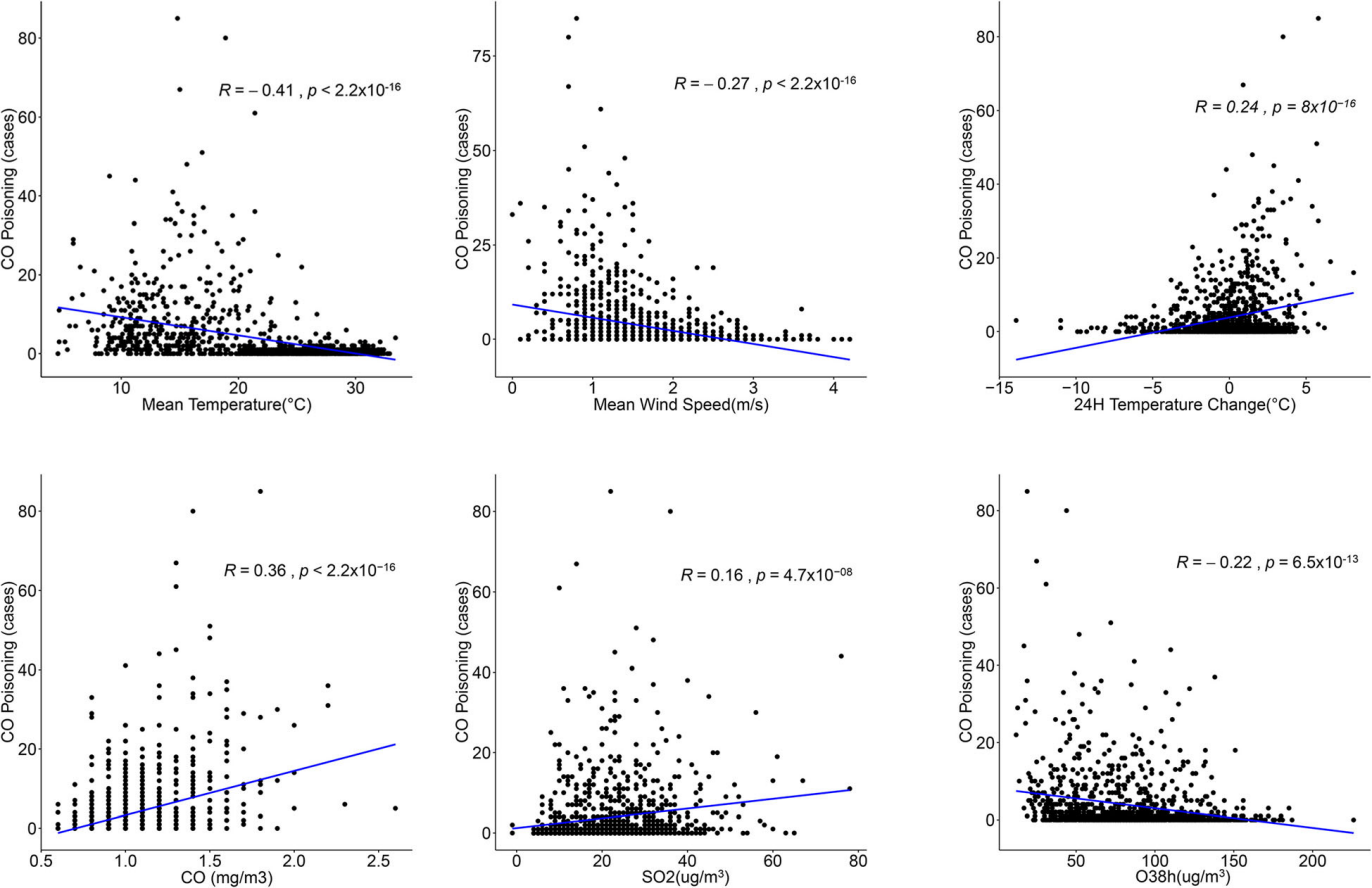
Logistic regression of CO poisoning by daily mean meteorological and pollutant factors. Abbreviation: CO, carbon monoxide; O_3_, ozone; SO_2_, sulfur dioxide

Logistic regression analyses of daily (Fig. 2) and monthly (Figure S1) CO poisoning levels were conducted separately, and the result showed that among the all parameters evaluated, CO poisoning was most closely associated with temperature, followed by air pressure and O_3_8h; among environmental factors, CO poisoning was most closely associated with CO concentration, followed by PM_10_.

### Ordinal logistic regression of CO poisoning risk levels and prediction model establishment

Results of our ordinal regression analyses showed that temperature (β: -0.193, OR: 0.82, 95% CI: 0.80, 0.85) was inversely associated with CO poisoning, whereas 24-h temperature difference (β: 0.221, OR: 1.25, 95% CI: 1.18, 1.31) and CO (β: 1.25, OR: 3.48, 95% CI: 2.82, 4.14) (all *P*s < 0.001) were positively associated with CO poisoning (Table 3). Wind speeds (β: -0.228, OR: 0.80, 95% CI: 0.54, 1.06; *P =* 0.086) and SO_2_ (β: -0.018, OR: 0.98, 95% CI: 0.96, 1.00; *P =* 0.058) were marginally associated with CO poisoning. Null associations were found for O_3_8h (β: 0.0008, OR: 1.00, 95% CI: 1.00, 1.01; *P =* 0.766). These results showed that CO poisoning was closely related with the ambient CO concentration, being 3.48 times more likely to occur along with per 1 mg/m3 increase in ambient CO concentrations and 1.25 times more likely to occur at per 1 °C increase in 24 h temperature change. In addition, we found that 0.82 times less possibility of CO poisoning occurred along with per 1 °C decrease in temperature.

**Table 3:** Ordinal logistic regression of CO poisoning admission by meteorological and environmental factors in total patients

Parameters	Coefficients	Std. Error	OR (95% CI)	*P-*values
Mean temperature, °C	−0.193	0.015	0.82 (0.80, 0.85)	< 0.001
Mean wind speed, m/s	−0.228	0.133	0.80 (0.54, 1.06)	0.086
24-h temperature change, °C	0.221	0.034	1.25 (1.18, 1.31)	< 0.001
CO, mg/m^3^	1.25	0.337	3.48 (2.82, 4.14)	< 0.001
SO_2_, ug/m^3^	−0.018	0.009	0.98 (0.96, 1.00)	0.058
O_3_8h, ug/m^3^	0.0008	0.003	1.00 (1.00, 1.01)	0.766
Intercepts				
1|2	−4.12	0.595	–	< 0.001
2|3	−2.93	0.584	–	< 0.001
3|4	−1.98	0.576	–	< 0.001
4|5	−0.92	0.576	–	0.108

Residual Deviance: 1747

AIC: 1767

Likelihood ratio tests was conducted for ordinal regression models (*P* < 0.001)

*Abbreviation*: *CO* Carbon monoxide, *O*_*3*_ Ozone, *SO*_*2*_ Sulfur dioxide

Based on the regression coefficient, prediction models for CO poisoning grades risk was established as follows: 1: using single meteorological factor as prediction factors and 2: using combined meteorological and pollutant factor as prediction factors. Single-factor and combined-factor prediction was conducted separately to fit a better model. A lower Akaike information criterion (AIC) value denotes a stronger model fit. Two fitting models were shown below:

1. logitPj = aj - 0.195 * temperature - 0.356 * wind speed + 0.224 * 24 h temperature change

1 $$ {\displaystyle \begin{array}{l}\mathrm{j}=1,2,3,4\\ {}\mathrm{a}1=-5.32,\mathrm{a}2=-4.15,\mathrm{a}3=-3.23,\mathrm{a}4=-2.20;\kern0.5em \mathrm{AIC}=1777\end{array}} $$

2: logitPj = aj - 0.193 * temperature - 0.228 * wind speed + 0.221 * 24 h temperature change + 1.25 * CO - 0.018 * SO_2_ + 0.0008 *O_3_8h;

2 $$ {\displaystyle \begin{array}{l}\mathrm{j}=1,2,3,4;\\ {}\mathrm{a}1=-4.12,\mathrm{a}2=-2.93,\mathrm{a}3=-1.98,\mathrm{a}4=-0.92;\kern0.5em \mathrm{AIC}=1767.\end{array}} $$

According to AIC standards, we identified that the combined-factor model with pollutant factors had a better goodness of fit than the single meteorological factor model.

### Examination of the CO poisoning prediction model

**Fig. 3:**
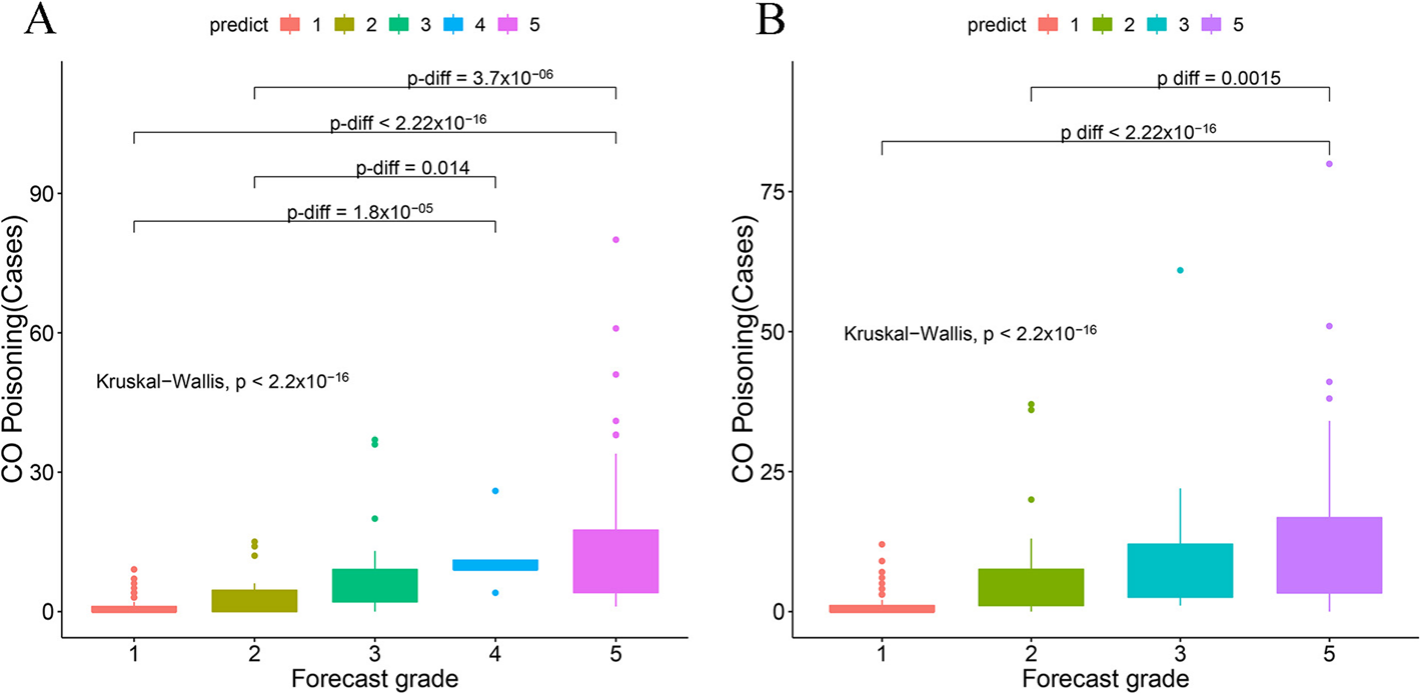
Median comparison of CO poisoning cases of different forecast grade. **a** Combing meteorological and environmental factors as prediction factor, **b** single meteorological factors as prediction factor

Prediction models were examined using actual CO poisoning levels in 2017 and the prediction potential were analyzed, which were shown in Fig. 3 and Additional file 1: Table S3. The boxplots showed that the median actual cases of CO poisoning gradually increased with predictive grades (*P*-diff < 0.001), i.e. the actual poisoning case number was lowest in grade 1 and increased gradually in grade 2–5 (Fig. 3). To assess the comprehensive prediction power of the models, we calculated average effective prediction rate in two extreme grades (Grade 1 & 5). Single-factor model showed an effective rate of 83.8% (average of 145/152 [Grade 1] and 47/65 [Grade 5]), slightly lower than that of 84.4% (average of 142/152 [Grade 1] and 49/65 [Grade 5]) in the combined-factor model. In addition, single-factor model predicted 0 days for Grade 4 and was presumed to be a little weaker in predicting serious poisoning grades compared with the combined model. Altogether, the single meteorological factors can predict the occurrence of CO poisoning cases, but the combined model with pollutant factors is more accurate and performs better especially for serious grades of CO poisoning.

## Discussion

Our data show that for the city of Liuzhou, meteorological and pollutant patterns of low temperatures, low wind speeds, low SO_2_ and high CO and O_3_8h levels were related to CO poisoning incidence. Among these, a prominent positive association between air CO and poisoning grades and an inverse association between temperature and poisoning grades were found. A prediction model based on those parameters can predict poisoning cases well, and the combined model with air pollutant factor are more accurate and perform better than models based on meteorological factors alone. Considering both meteorological and pollutant parameters as prediction factors will thus play a role in the prevention of CO poisoning.

Some previous studies have reported that domicile-related CO poisoning mainly occurred in cold months and at lower temperatures. Climate conditions influence air pollutant distributions. Cases of CO poisoning was severe worldwide. However, few studies have investigated the association between CO poisoning and climatic factors such as wind speed, daily temperatures and air pollutants [6, 10]. A population-based case-control study of Taiwan found a 2.15-fold increase in CO poisoning mortality in cold periods with maximum daily temperatures of less than 18.4 °C than in warm periods with daily maximum temperatures of 27.1 °C or greater [10]. The onset of colder weather typically results in behavior changes (e.g., higher levels of indoor heating). Xie J et al. found that wind speed was the main factor that affects CO poisoning accidents which occurred in dwellings and that ventilation, smoke emissions, differences between indoor and outdoor temperatures, low daily gas pressure changes could aggravate CO poisoning occurrence [11]. In addition, low wind velocities, ground-level inversion and fog aggravate domicile-derived CO poisoning, as they impede the rise of combustion emissions through chimneys and the dispersal of indoor CO [7, 12]. CO poisoning outbreaks are also related to storms and power outages [13].

We consistently found temperatures to be the most important protection against CO poisoning along with wind speed. Climate conditions were also found to be closely related to air pollutants. Low temperatures and wind speeds lead to limited air movements, potentially increasing concentrations of air pollutants. A series of studies have shown that ambient air pollutants SO_2_, CO, O_3_, PM_10_, PM_2.5_, and NO_2_ can induce a variety of acute symptoms [14] and are related to hospitalizations for respiratory [15, 16] and cardiovascular diseases [17, 18]. Notably, CO is reportedly strongly associated with mortality and heart failure [19]. While few studies have explored the influence of air pollutants on CO poisoning, except one study found a poor  pollutant distribution on days with poisoning events [7]. Epidemiological studies have also shown that air pollutants contribute to daily mortality [20] and that the association remains even at very low pollution levels [21]. The specific effects of air CO and other pollutants on the incidence of CO poisoning are unknown. Our data show strong independent positive effects of pollutant (SO_2_, CO and O_3_8h) distributions and CO poisoning. In particular, a 3.48-fold higher risk of CO poisoning was found for per 1 mg/m3 increase in air CO concentration (OR [95% CI], 3.48 [2.82, 4.14], *P* < 0.001). Among underlying mechanisms involved, it was assumptive that high outdoor CO concentrations might impede the diffusion of indoor CO gas. To our knowledge, no other study has investigated the correlation between ambient CO concentration and indoor CO poisoning. Future studies must clarify underlying mechanisms involved.

The exposure dose of CO depends on its air concentration, the exposure time and the patient’s breathing volume per minute. Indoor smoke elevates baseline carboxyhemoglobin levels [22, 23]. Other indoor sources include exhaust from poorly ventilated gas- and oil-burning home appliances and the infiltration of outdoor pollutants [24]. In unpolluted ambient air, concentrations of CO range from 0.02–1.0 ppm; however, in urban areas, CO concentrations can increase 10-fold in periods of atmospheric stagnation [25, 26] (i.e., during temperature inversions in winter or when sedentary air masses form in summer). CO levels of approximately 100 ppm can be detected in kitchens when a gas stove is used for cooking or when there is a gas leak [24]. The Chinese government recommends that the indoor CO concentration not exceed a maximum of 50 ppm. Hence, preventing CO leaks and reducing the ambient CO concentration are central to the prevention of CO poisoning.

A single factor may not account for the comprehensive causes of CO poisoning. Previous studies have examined the effects of meteorological change on CO poisoning, and our data show that pollutant-related factors might also play an important role in CO poisoning incidence. Meteorological and pollutant patterns—low air temperatures, wind speeds, and SO_2_ levels and high 24-h temperature differences, CO levels and O_3_8h concentrations—might increase the incidence of domicile-related CO poisoning. In this study, we established two prediction models: a meteorological model covering temperature, 24 h temperature changes, and wind speed and a model covering meteorological and pollutant-related factors (SO_2_, CO and O_3_8h). Both models effectively predict CO poisoning occurrence. The combined model is superior to the single factor model in the following respects. First, the combined model is more accurate when applied to extreme grades (84.4%, which is slightly higher than the value of 83.8% found for the single-factor model). Second, the combined-factor model is more effective at predicting serious poisoning grades than the single-factor model. Third, the single-factor model cannot identify cases of level 4 poisoning. These results show that both meteorological and air pollutant parameters must be considered for the prediction of CO poisoning [12]. Future studies must validate these findings and explore more underlying mechanisms through which outdoor environmental conditions influence CO poisoning. In addition, high-risk populations (e.g., the elderly and children) must be closely considered.

CO poisoning is preventable and avoidable. Automobiles and cooking and heating appliances have long been regarded as the main environmental sources of CO [27]. Gas stoves and water heaters are very popular in the city of Guangxi. A gas leak due to improper installation or aging parts can result in CO poisoning. Thus, safety education designed to increase awareness of CO poisoning and of ways to reduce CO production should be effective at reducing the occurrence of domicile-related poisoning [28, 29]. Public service warnings and government supervision can also help prevent poisoning events, especially during storms [30]. As we found that combined-factor prediction shows strong prevention potential, incorporating CO poisoning predictions into weather broadcast programs could raise public awareness of CO poisoning occurrence [7]. Effective air purification measures taken by environmental protection agencies could also improve the prevention of CO poisoning, and installing CO detectors in residences have favorable cost benefits [3], which can be used as an important secondary prevention measure [31]. Finally, prompt treatment measures including fast and correct diagnosis and effective therapy should decrease the incidence of death from CO poisoning [30].

This study presents some strengths. First, we are the first to analyze the influence of pollutants on CO poisoning and to establish a prediction model of CO poisoning based on meteorological factors alone and on a combination with environmental parameters. Second, in applying our models to actual cases of CO poisoning, we found that the model considering both meteorological and pollutant parameters can predict CO poisoning well and performs better than the model based on meteorological factors alone in its accuracy and predictive potential for high poisoning grades. This study also has some limitations. First, the medical records considered in this study represent cases of CO poisoning in the studied region but not include cases with missed diagnoses, patients who died close to sources of CO poisoning and were not admitted to a designated hospital. Nevertheless, cases of CO poisoning considered in this study were collected from four designated hospitals for CO poisoning, which ensured a more comprehensive and reliable dataset. Second, while we established a prediction model based on multiple meteorological and environmental factors and validated the model in several ways, the model only xaccounts for part etiological causes of CO poisoning.

## Conclusions

Temperatures, wind speeds, daily temperature changes, ambient SO_2_, CO and O_3_8h serve as significant predictors of CO poisoning. The proposed prediction models based on meteorological and pollutant parameters can help prevent CO poisoning by targeting high-risk populations. The developed model considering both meteorological and pollutant parameters offers superior predictive power than the proposed single-factor model, especially for days of high poisoning risk. Future studies must validate our findings.

## Methods

### Population and data source

CO poisoning data for 2015–2017 were collected from the emergency department of four hospitals which were designated by Liuzhou Health Commission for CO poisoning. The medical records of all eligible patients were reviewed, and cases of suicide and attempted suicide and accidental CO poisoning were excluded. Meteorological and pollutant data for the same period were obtained from the Liuzhou Meteorological Bureau and Liuzhou Environmental Protection Agency, respectively. All meteorological and pollutant parameters were taken from the 6 representative monitoring sites.

### CO poisoning gradation

To construct a prediction model of CO poisoning, we graded CO poisoning into 5 levels according to the number of daily cases of CO poisoning as follows: Level 1, 0 cases of CO poisoning daily; Level 2, 1 case of CO poisoning daily; Level 3, a moderate risk of daily CO poisoning with > 1 and ≤ 3 cases; Level 4, a high risk of daily CO poisoning with > 3 and ≤ 7 cases; and Level 5, a very high risk of daily CO poisoning with > 7 cases.

### Data and statistical analysis

First, to investigate possible correlations between the occurrence of CO poisoning and meteorological and pollutant parameters, records of meteorological (temperature, gas pressure and daily changes, humidity, wind direction, and wind speed) and air pollutant parameters (sulfur dioxide (SO_2_), nitrogen dioxide (NO_2_), inhalable particulate matter (PM)10, PM_2.5_, CO, and ozone (O_3_8h)) for Liuzhou for 2015–2017 were collected. Mean daily meteorological and pollutant parameters and cases of CO poisoning were analyzed.

Further, Pearson correlation analyses were performed to examine the correlation between meteorological and pollutant factors and CO poisoning. Partial correlation analyses were conducted with related parameters controlled. Indices of meteorological and pollutant parameters showing a significant partial correlation (*P* < 0.05) with CO poisoning were selected as prediction factors. Logistic regression was used to evaluate the association between CO poisoning levels and daily and monthly mean values of the single prediction factors. Multivariable ordinal logistic regression was used to evaluate coefficients and odds ratios (95% CI) per 1 unit increase in the predictive factors based on data for 2015 and 2016. Then, the coefficient of predictors obtained was used to establish a prediction model of CO poisoning.

We used data for 2017 to examine the predictive power of both models. From the probability model established, we obtained predictive CO poisoning risk levels for each day of 2017 based on intraday meteorological and pollutant information and in turn determined the most likely risk levels. The Kolmogorov-Smirnov test was used to examine differences in average cases of CO poisoning based on pairwise prediction grades. All analyses were conducted using R3.5 (https://www.r-project.org/) where a 2-tailed probability of less than 0.05 was considered statistically significant.

## Supplementary Information

**Additional file 1: Table S1.** Distribution of daily incidence counts and overall daily pollutant and meteorological measures. **Table S2.** Pearson correlation between CO poisoning admission and pollutant parameters. **Figure S1.** Logistic regression of CO poisoning by monthly mean meteorological and pollutant factors. **Table S3.** A comparison of actual CO poisoning level in 2017 and predictive CO poisoning grade based on the combination of meteorological and pollutant factors.

## Data Availability

The data access committee comprises the first author and the corresponding author, and there is no restriction to data access. De-identified individual-level data are available upon reasonable request to H.L.R at drruanhailin@163.com.

## Abbreviations

CO: Carbon monoxide
O_3_: Ozone
PM_10_: Particle matter 10
PM_2.5_: Particle matter 2.5
NO_2_: Nitrogen dioxide
SO_2_: Sulfur dioxide
