# Supplementary material for: Carbon monoxide poisoning: a prediction model using meteorological factors and air pollutant
Source: BMC Proc. 2021 Mar 2;15(Suppl 1):1. doi: 10.1186/s12919-021-00206-7 (PMC7923450; doi:10.1186/s12919-021-00206-7)
Supplement: Supplementary file 1 — Additional file 1: Table S1. Distribution of daily incidence counts and overall daily pollutant and meteorological measures. Table S2. Pearson correlation between CO poisoning admission and pollutant parameters. Figure S1. Logistic regression of CO poisoning by monthly mean meteorological and pollutant factors. Table S3. A comparison of actual CO poisoning level in 2017 and predictive CO poisoning grade based on the combination of meteorological and pollutant factors. [file 12919_2021_206_MOESM1_ESM.docx]

Additional information

Carbon monoxide poisoning: a prediction model using meteorological factors and air pollutant

Hai-Lin Ruan^1^, Wang-Shen Deng^1^, Yao Wang^1^, Jian-Bing Chen^2^, Wei-Liang Hong^3^, Shan-Shan Ye^1^, Zhuo-Jun Hu^4*^

**Table S1.** Distribution of daily incidence counts and overall daily pollutant and meteorological measures.

|  | Min | 10% | 50% | 90% | Max | Mean (SD) |
| --- | --- | --- | --- | --- | --- | --- |
| Mean temperature, ℃ | 3.6 | 11.2 | 23.4 | 30.1 | 33.4 | 21.7 (7.19) |
| 24-hour temperature change, ℃* | 0.0 | 0.20 | 1.20 | 3.60 | 13.9 | 1.66 (1.59) |
| Mean atmospheric pressure, hPa | 983 | 992 | 1001 | 1013 | 1028 | 1002 (7.66) |
| 24-hour atmospheric pressure change, hPa* | 0.0 | 0.30 | 1.60 | 4.59 | 13.6 | 2.16 (1.92) |
| Humidity, % | 28.0 | 58.7 | 73.5 | 88.2 | 99.0 | 73.4 (11.5) |
| Maximum wind direction, ° | 23 | 23 | 180 | 338 | 360 | 175 (115) |
| Mean wind speed, m/s | 0.0 | 0.83 | 1.40 | 2.27 | 4.20 | 1.48 (0.60) |
| SO_2_, ug/m^3^ | 0 | 8.00 | 19.00 | 50.00 | 228 | 24.8 (19.3) |
| NO_2_, ug/m^3^ | 0 | 11.00 | 23.00 | 45.00 | 131 | 26.1 (14.6) |
| PM_10_, ug/m^3^ | 0 | 29.00 | 63.00 | 142.00 | 403 | 75.0 (47.6) |
| CO, mg/m3 | 0 | 0.68 | 1.00 | 1.52 | 3.6 | 1.05 (0.42) |
| O_3_8h, ug/m^3^ | 0 | 49.00 | 100.00 | 164.00 | 386 | 87.5 (40.5) |
| PM_2.5_, ug/m^3^ | 0 | 19.0 | 43.5 | 101.0 | 333 | 53.1 (36.8) |

Abbreviation: CO, carbon monoxide; NO_2_, nitrogen dioxide; SO_2_, sulfur dioxide; O_3_8h, ozone; PM, particulate matter.

Data are shown as partial correlation coefficient (significance).

* The value was calculated as difference on the second day minus the value on the first day.

**Table S2.** Pearson correlation between CO poisoning admission and pollutant parameters.

|  | SO_2_ | NO_2_ | PM_10_ | CO | O_3_8h | PM_2.5_ | CO poisoning cases |
| --- | --- | --- | --- | --- | --- | --- | --- |
| SO_2_ | 1 |  |  |  |  |  |  |
| NO_2_ | 0.487† | 1 |  |  |  |  |  |
| PM_10_ | 0.571† | 0.822† | 1 |  |  |  |  |
| CO | 0.477† | 0.608† | 0.501† | 1 |  |  |  |
| O_3_8h | 0.179† | 0.146† | 0.404† | -0.156† | 1 |  |  |
| PM_2.5_ | 0.568† | 0.777† | 0.960† | 0.571† | 0.271† | 1 |  |
| CO poisoning cases | 0.164† | 0.371† | 0.296† | 0.353† | -0.212† | 0.338† | 1 |

Abbreviation; CO, carbon monoxide; NO_2_, nitrogen dioxide; SO_2_, sulfur dioxide; O_3_, ozone; PM, particulate matter.

†P-value for correlation < 0.001.

‡P-value for correlation between 0.001 and 0.05.


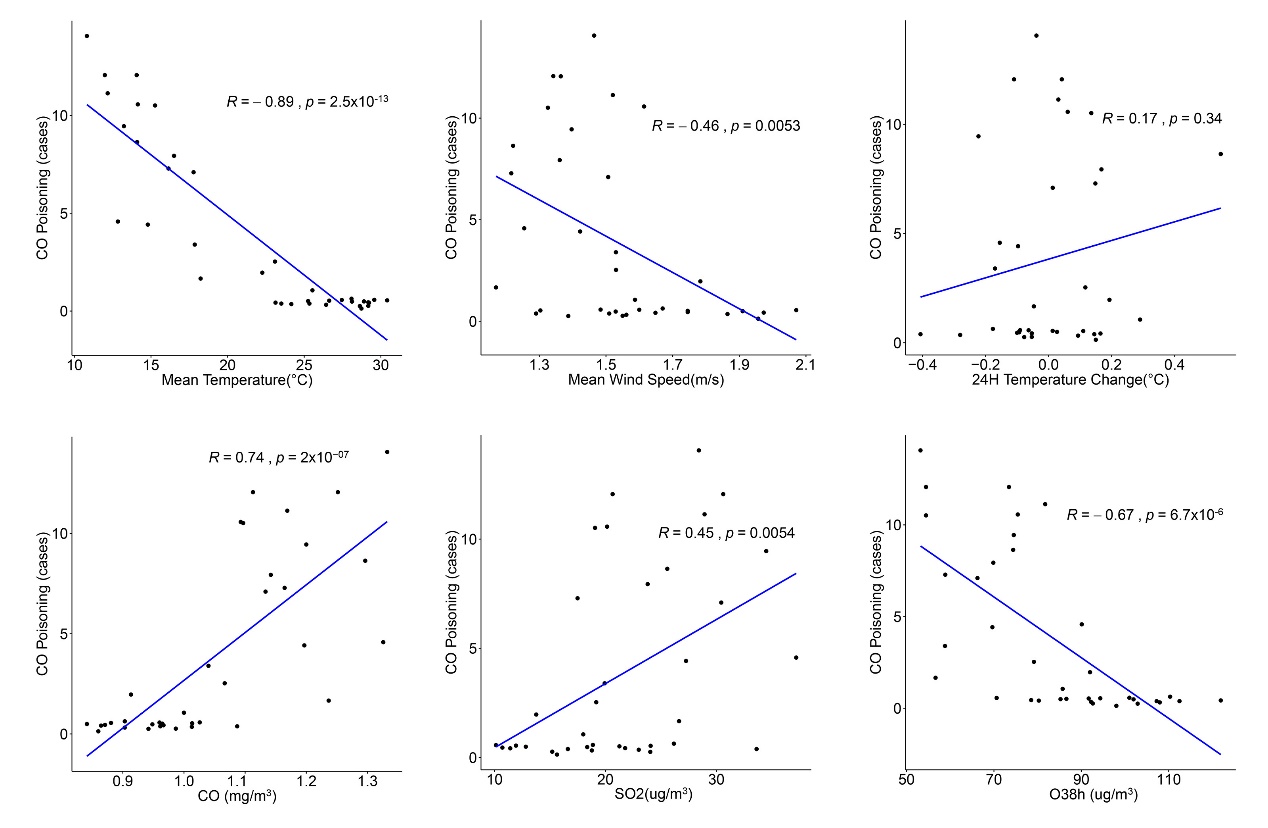


**Figure S1. Logistic regression of CO poisoning by monthly mean meteorological and pollutant factors.**

Abbreviation; CO, carbon monoxide; NO_2_, nitrogen dioxide; SO_2_, sulfur dioxide; O_3_8h, ozone; PM, particulate matter.

Table S3 A comparison of actual CO poisoning level in 2017 and predictive CO poisoning grade based on the combination of meteorological and pollutant factors.

| Actual CO poisoning | Prediction grade based on single meteorological factors | | | | | | Prediction grade based on the combined meteorological and pollutant factors | | | | | |
| --- | --- | --- | --- | --- | --- | --- | --- | --- | --- | --- | --- | --- |
|  | Grade 1 | Grade 2 | Grade 3 | Grade 4 | Grade 5 | Total | Grade 1 | Grade 2 | Grade 3 | Grade 4 | Grade 5 | Total |
| 1, case number = 0 | 145 | 5 | 0 | 0 | 2 | 152 | 142 | 8 | 2 | 0 | 0 | 152 |
| 2, case number = 1 | 54 | 3 | 3 | 0 | 9 | 69 | 55 | 2 | 3 | 0 | 9 | 69 |
| 3, case number >1 and ≤3 | 21 | 7 | 4 | 0 | 11 | 43 | 21 | 4 | 8 | 0 | 10 | 43 |
| 4, case number >3 and ≤7 | 9 | 6 | 4 | 0 | 17 | 36 | 9 | 6 | 5 | 1 | 15 | 36 |
| 5, case number >7 | 3 | 7 | 8 | 0 | 47 | 65 | 2 | 3 | 7 | 4 | 49 | 65 |
| Total | 232 | 28 | 19 | 0 | 86 | 365 | 229 | 23 | 25 | 5 | 83 | 365 |

Abbreviation: CO, carbon monoxide.
